# Tuneable on-demand single-photon source in the microwave range

**DOI:** 10.1038/ncomms12588

**Published:** 2016-08-22

**Authors:** Z. H. Peng, S. E. de Graaf, J. S. Tsai, O. V. Astafiev

**Affiliations:** 1 Physics Department, Royal Holloway, University of London, Egham, Surrey TW20 0EX, UK; 2 Center for Emergent Matter Science, RIKEN, Wako, Saitama 351-0198, Japan; 3 National Physical Laboratory, Teddington TW11 0LW, UK; 4 Department of Physics, Tokyo University of Science, Kagurazaka, Tokyo 162-8601, Japan; 5 Moscow Institute of Physics and Technology, Dolgoprudny 141700, Russia

## Abstract

An on-demand single-photon source is a key element in a series of prospective quantum technologies and applications. Here we demonstrate the operation of a tuneable on-demand microwave photon source based on a fully controllable superconducting artificial atom strongly coupled to an open-ended transmission line. The atom emits a photon upon excitation by a short microwave π-pulse applied through a control line. The intrinsically limited device efficiency is estimated to be in the range 65–80% in a wide frequency range from 7.75 to 10.5 GHz continuously tuned by an external magnetic field. The actual demonstrated efficiency is also affected by the excited state preparation, which is about 90% in our experiments. The single-photon generation from the single-photon source is additionally confirmed by anti-bunching in the second-order correlation function. The source may have important applications in quantum communication, quantum information processing and sensing.

## Abstract (web summary)

Microwave single photon sources are important for quantum applications, but their design often incorporates a resonator that fixes the frequency of the emitted photon. Here, the authors demonstrate a tuneable on-demand photon source based on an artificial atom asymmetrically coupled to two transmission lines.

## Graphical abstract

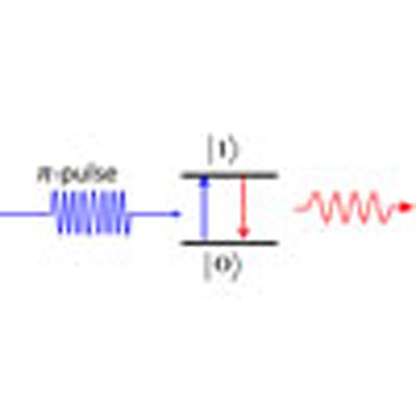

Control and manipulation with light at the single-photon level^1^,^2^,^3^,^4^ is interesting from fundamental and practical viewpoints. In particular, on-demand single-photon sources are of high interest because of their promising applications in quantum communication, quantum informatics, sensing and other fields. In spite of several realizations in optics^5^,^6^,^7^,^8^,^9^, practical implementation of photon sources imposes a number of requirements, such as high photon generation, collection efficiencies and frequency tunability. Recently developed superconducting quantum systems provide a novel basis for the realization of microwave (MW) photon sources with photons confined in resonator modes^10^,^11^,^12^,^13^,^14^,^15^, which is essentially different from the three-dimensional (3D) case of optical photon sources^16^. However, all the circuits consist of two elements (the resonator and the quantum emitter system) and the generated photon frequency is fixed to the resonator frequency.

In this work, we propose and realize a different approach: a single-photon source based on a tuneable artificial atom coupled asymmetrically to two open-ended transmission lines (one-dimensional (1D) half-spaces) and a similar scheme was proposed by Lindkvist *et al*.^17^. The atom is excited from a weakly coupled control line (*c*) and emits a photon to a strongly coupled emission line (*e*). The photon freely propagates in the emission line and can be further processed using, for example, nonlinear circuit elements. Among the advantages of our circuit is its simplicity: it consists of a single element.

## Results

### Operation principles and device description

**Figure 1:**
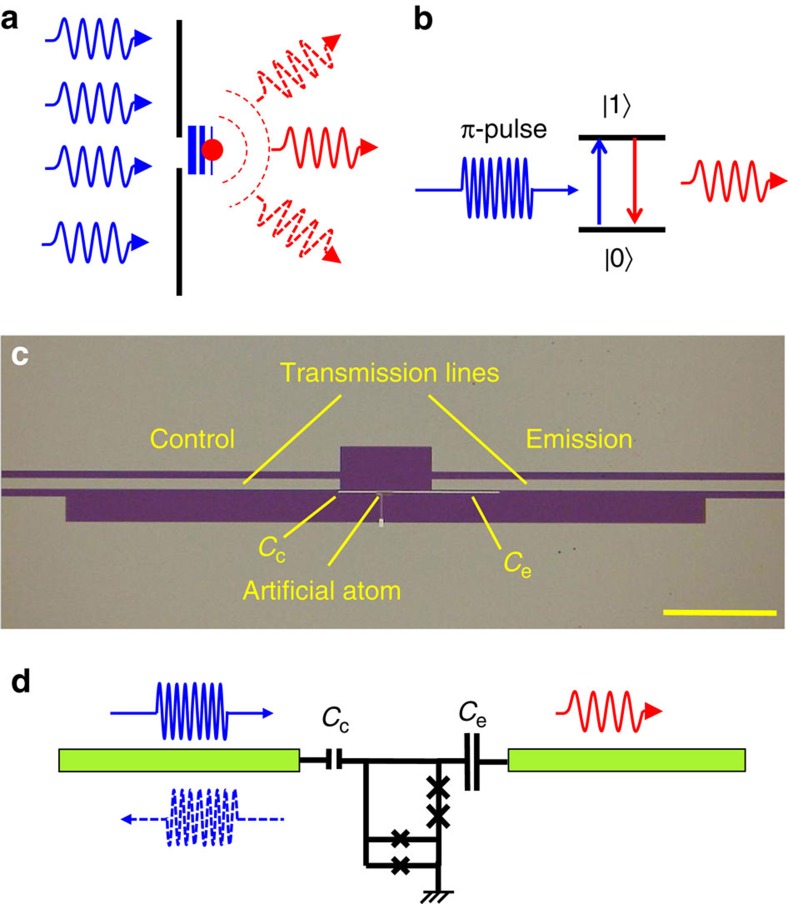
The single-photon source. (**a**) Optical analogue of the source. A non-transparent screen with a hole, much less than a wavelength, forms two half-spaces. A two-level atom is situated in the right subspace close to the hole. The incident light from the left-hand side excites the atom by evanescent waves, which, however, cannot penetrate through the hole. The excited atom, in turn, emits radiation mainly into the right sub-space. (**b**) Mechanism of the single-photon generation. The atom exited by a *π*-pulse (blue) of the incident radiation relaxes with a photon emission into the right sub-space (denoted by red colour). (**c**) Optical micrograph of the device. The artificial atom is in the middle and the thin long metallic line from the atomic loop forms capacitances between the atom and the control/emission transmission lines. The scale bar in the bottom-right side is 100 μm. (**d**) An equivalent electrical circuit of the photon source. A superconducting loop with two junctions and an α-loop at the bottom forms the tuneable two-level quantum system. The system is coupled to the control and emission lines by capacitances *C*_c_ and *C*_e_, respectively.

An optical analogue of the proposed single-photon source consists of a two-level atom situated near a tiny hole (much smaller than the wavelength) in a non-transparent screen (Fig. 1a). The atom is slightly shifted towards the right-hand-side space, defining an asymmetric coupling to the half-spaces. By applying powerful light from the left side, the atom can be excited by evanescent waves, which cannot propagate in the right-hand-side space because of their rapid decay. On the other hand, the excited atom emits photons into the right-hand-side space (Fig. 1b). In practice, the presented layout is difficult to build using natural atoms and, even if one succeeds, another problem must be solved: the low collection efficiency of emitted photons in the 3D space. These problems can be easily avoided by using on-chip superconducting quantum circuits coupled to 1D transmission lines^18^,^19^,^20^. Figure 1c shows a circuit with an artificial atom coupled asymmetrically to a pair of open-ended coplanar transmission lines (1D-half spaces), each with *Z*=50 Ω impedance. The coupling capacitances *C*_c_ and *C*_e_ are between the artificial atom and the control and emission lines, respectively (shown on the equivalent circuit in Fig. 1d). The capacitances can be approximated as point-like objects because their sizes are much smaller than the wavelength of the radiation (∼1 cm). Note also that the transmission lines in the centre of our device (about 80 μm for each line) slightly differ from 50 Ω because of the shifted down ground plane; however we can ignore it because they are also much shorter than the wavelength. A MW pulse is applied from the control line, exciting the atom, and then the atom emits a photon mainly to the emission line because of asymmetric coupling: *C*_e_/*C*_c_≈30. The following are intrinsic features of the device: the two lines are well isolated from each other so that the excitation pulse does not leak from the control line to the emission line; because of the strong asymmetry, the excited atom emits a photon with up to 1−(*C*_c_/*C*_e_)^2^ output probability; the photon is confined in the 1D transmission line and can be easily delivered to other circuit elements through the line.

The artificial atom schematically shown in Fig. 1d is a controllable two-level system based on a tuneable gap flux qubit^21^,^22^,^23^,^24^, that is coupled to two Nb coplanar lines. The atom is fabricated by Al/AlOx/Al shadow evaporation techniques. It contains two identical junctions in series implemented in the loop together with a dc-SQUID (called an *α*-loop), shown in the bottom part of the device in Fig. 1d. Here *α*≈0.7 specifies the nominal ratio between the two critical currents in the dc-SQUID and the other two Josephson junctions in the loop. The magnetic fluxes are quantized in the loop: an integer number, *N*, of the magnetic flux quanta, Φ_0_, can be trapped. At the magnetic fields where the induced magnetic flux in the loop is equal to Φ=Φ_0_(*N*+1/2), two adjacent flux states |0〉 and |1〉 with *N* and *N*+1 flux quanta, which is corresponding to oppositely circulating persistent currents, are degenerated. The degeneracy is lifted because of the finite flux tunnelling energy Δ_*N*_, determined by the effective dc-SQUID Josephson energy and varies between different degeneracy points (depends on *N*). The energy splitting of the atom 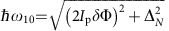 is controlled by fine adjustment of the magnetic field *δ*Φ in the vicinity of the degeneracy points, where *δ*Φ=Φ−(*N*+1/2)Φ_0_ and *I*_p_ is the persistent current in the main loop. (We neglect the weak dependence of Δ_*N*_ on *δ*Φ.)

The capacitances of the circuit are estimated to be *C*_c_≈0.3 fF and *C*_e_≈9 fF. The effective impedance between the two lines because of the capacitive coupling is *Z*_C_=1/*iω*(*C*_c_+*C*_e_), which is about 2 kΩ for *ω*/2*π*=10 GHz and the transmitted part of the power is as low as |2*Z*/*Z*_C_|^2^≈2.5 × 10^−3^. This enables nearly perfect line decoupling.

### Device characterisation

**Figure 2:**
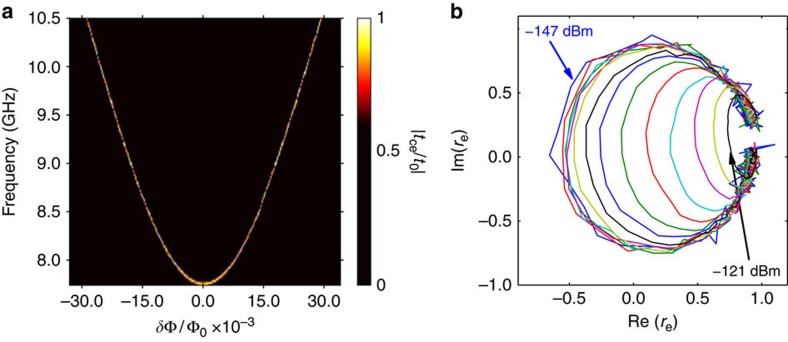
Response of the system to the continuos microwaves. (**a**) Transmission spectrum through the artificial atom. The normalized transmission amplitude |*t*_ce_/*t*_0_| from the control line to the emission line versus flux bias *δ*Φ, measured from half-flux quantum Φ_0_/2, where the energy is minimal. The transmission is suppressed everywhere except at the resonance frequencies of the atom. At the resonance frequency, the atom, excited from the control line, reemits the radiation to the emission line. (**b**) Reflection coefficient *r*_e_ in the emission line around *δ*Φ=0 (the minimal atomic energy) on a complex plane. Reflection coefficient *r*_e_ is plotted in real-imaginary coordinates for probing powers from −147 to −121 dBm with a step of 2 dB.

Our experiment is carried out in a dilution refrigerator at a base temperature of around 30 mK. We first characterize our device by measuring the transmission coefficient *t*_ce_ from the control line to the emission line using a vector network analyser (VNA) and the reflection coefficient *r*_e_ from the emission line. Figure 2a shows a two-dimensional (2D) plot of the normalized transmission amplitude |*t*_ce_/*t*_0_| in the frequency range 7.75–10.5 GHz with the magnetic flux bias *δ*Φ from −30 to 30 mΦ_0_ around the energy minimum, where *t*_0_ is the maximal transmission amplitude. The transmission is suppressed everywhere except in the narrow line that corresponds to the expected atomic resonance at *ω*_10_ and is a result of the photon emission from the continuously driven atom. The spectroscopic curve is slightly asymmetric with respect to *δ*Φ=0 because of the weak dependence of Δ_*N*_ on *δ*Φ. From the spectroscopy line we deduce the parameters of the two-level system: the tunnelling energy is Δ=min(*ħω*_10_)=*h* × 7.750 GHz at *δ*Φ=0 and the persistent current in the loop is *I*_p_≈45 nA.

To evaluate the coupling of our atom to the emission line, we also measure the reflection at *δ*Φ=0 with different probing powers from −147 to −121 dBm. Next, Fig. 2b shows the reflection coefficient *r*_e_ mapped in the complex plain, measured in the case of atom excitation from the emission line (opposite to the case of source operation). The curve changes its form from circular to oval, which reflects the transition from the linear weak-driving regime up to the nonlinear strong-driving regime of the two-level system^18^.

We next derive the dynamics of the point like atom (the loop size ∼10 μm is much smaller than the wavelength *λ*∼1 cm) located at *x*=0 and coupled to the 1D open space via an electrical dipole. We also take into account the fact that 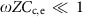. The atom is driven by the oscillating voltage at the frequency ω of the incident wave *V*_0_(*x*, *t*)=*V*_0_*e*^−*iωt*+*ikx*^ in the control line and the resulting driving amplitude of 2*V*_0_ cos *ωt*=Re[*V*_0_*e*^−*iωt*+*ikx*^+*V*_0_*e*^−*iωt*−*ikx*^]|_*x*=0_ is the sum of the incident and reflected waves. The Hamiltonian of the atom in the rotating-wave approximation is *H*=−(*ħδωσ*_*z*_+*ħ*Ω*σ*_*x*_)/2, where *δω*=*ω*−*ω*_10_ and *ħ*Ω=−2*V*_0_*C*_c_*ν*_a_ with the electric dipole moment of the atom *ν*_a_ (between *C*_c_ and *C*_e_). The atomic voltage creation/annihilation operator is 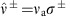, where 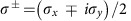. The driven atom generates voltage amplitudes of *V*_c,e_(*t*)/2=*iωZC*_c,e_*ν*_*a*_〈*σ*^−^〉*e*^−*iωt*^ in the control (*x*<0) and emission (*x*>0) lines. Substituting the relaxation rates 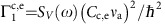 due to voltage quantum noise (*S*_*V*_(*ω*)=2*ħωZ*) from the line impedance *Z* in each line, we obtain


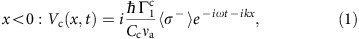



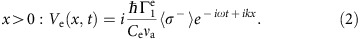


In the ideal case of suppressed pure dephasing (*γ*=0) and in the absence of nonradiative decay 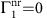, the power ratio between the control and emission lines generated by the atom under resonance is 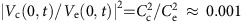, which means that up to 99.9% of the power generated by the atom can be emitted into the emission line. This allows us to measure the spectroscopy curve shown in Fig. 2a. To find 〈*σ*^−^〉 under continuous driving, we solve the master equation by considering the total relaxation rate 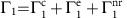, where 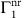 is the nonradiative relaxation rate (for a photon absorbed by the environment). Here, the dephasing rate is Γ_2_=Γ_1_/2+*γ*, where γ is the pure dephasing rate. The solution is 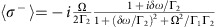. The reflection in the control line and the transmission coefficient from the control line to the emission line are *r*_c_=1+*V*_c_(0, *t*)/*V*_0_(0, *t*) and *t*_ce_=*V*_e_(0, *t*)/*V*_0_(0, *t*), respectively. At the weak-driving limit 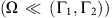,


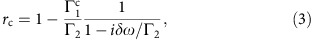



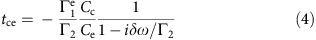


are circular plots in the complex plane. Similar to equation (3), we can write down the following expression for the reflection in the emission line


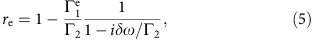


with the substitution 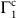 by 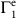. We will further use this expression to characterize the coupling strength and efficiency of our device.

Furthermore, the excited atom emits an instantaneous power proportional to the atomic population *I*_1_(*t*) (ref. 19) that can be straightforwardly expressed as


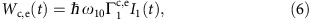


where *I*_1_=(1−〈*σ*_*z*_〉)/2. If the atom is prepared in the excited state |1〉 at *t*=0, the probability decays according to *I*_1_(*t*)=exp(−Γ_1_*t*). In addition, the efficiency of the photon emission to the right line is 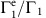, which ideally can be as high as 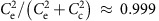. The plot in Fig. 2b gives us a measure of the coupling strength of the atom to the emission line. Using equation (5), we estimate the highest possible efficiency of photon generation to be 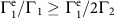
=0.79. Note that in real experiments it is additionally affected by the excited state control efficiency because of the competition between excitation and relaxation processes. However, the manipulation efficiency is not fundamentally limited and can be nearly one, if the available equipment allows to make π-pulse lengths much shorter than 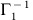.

### Device operation

**Figure 3:**
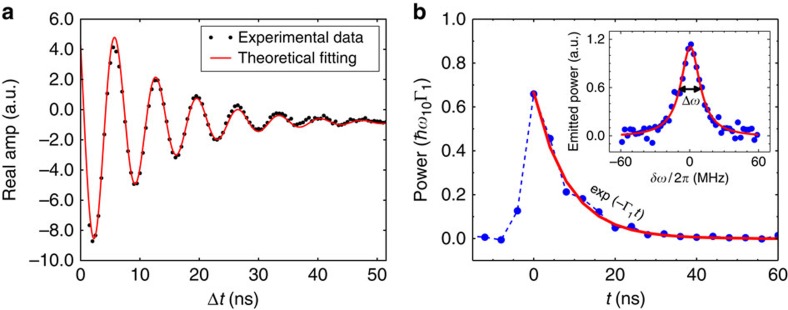
Device operation. (**a**) Rabi oscillations of the two-level atom coupled to the two half-spaces measured by VNA. The atom is excited by MW pulses of length Δ*t* from the control line with repetition time *T*=80 ns, and the coherent emission is detected from the emission line. (**b**) The emitted photon shape normalized to the single photon power. The red solid curve shows a fit to exp(−Γ_1_*t*). The inset demonstrates an emission peak (blue dots), when a π-pulse is applied at Δ*t*_*π*_ fitted by a red Lorentzian curve (Δ*ω*=Γ_1_).

Our photon source based on the conversion of an atomic excitation into a MW photon requires efficient control of the quantum states. Figure 3a shows measured quantum oscillations. We monitor the coherent emission from the atom into the emission line by VNA when a train of identical excitation MW pulses, each of length Δ*t* with period *T*=80 ns, is applied from the control line. The amplitude of the emission oscillates with Δ*t*. The maxima and minima of the oscillations correspond to |〈*σ*^±^〉|≈±1, when the atom is in the maximally superposed states with 50% population. For the single-photon source operation, we tune the pulse length to obtain the maximum incoherent emission (defined as a π-pulse and its length is Δ*t*_*π*_=3.5 ns), emitting a single photon from the atom excited state in every pulse period. The traces are then amplified and digitized with sampling time 4 ns. The traces *V*(*t*) of repeated measurements are then squared and accumulated. The typical photon shape *P*(*t*) obtained after 2 × 10^9^ times averaging is shown in Fig. 3b. The inset shows the averaged emission power peak excited by the *π*-pulses with repetition time and measured by a spectrum analyser. Using the Lorentzian fit, we obtain a FWHM Δ*ω*/2*π*≈20 MHz, which is equal to the relaxation rate Γ_1_.

### Measurements of correlation functions

The two-level system operation together with the Rabi oscillations prove that the source generates a single photon at a time. Nevertheless, we provide additional evidence of the single-photon generation by measuring the second-order correlation function with linear detectors (microwave amplifiers)^25^. Such a demonstration is straightforward in optics due to existence of photon counters but extremely demanding in the microwave range, where the microwave amplifiers have typical signal-to-noise ratio in the single-photon regime is less than 10^−2^ in power and, therefore, long accumulation of statistics is required.

**Figure 4:**
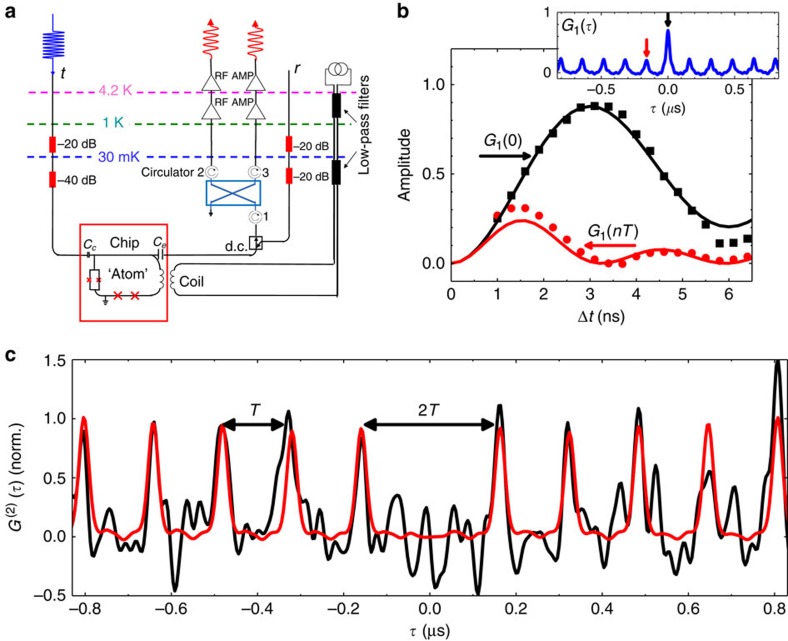
Correlation function measurements. (**a**) The experimental setup for Hanbury–Brown–Twiss measurements with linear detectors in the microwave frequency domain. (**b**) The first-order correlation function dynamics. The inset shows typical 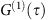. The black squares and red dots represent experimentally measured dynamics of central peak *G*^(1)^(0) and side peaks *G*^(1)^(*nT*), respectively. The solid curves show simulations with the actual device parameters, including dissipation. (**c**) The black curve is measured second-order correlation function 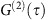 and the red one represents the simulated second-order correlation function using the measured photon traces. The second-order correlation function is a result of averaging of 1.5 × 10^10^ traces with 1,600 points in each trace with 4 ns sampling rate. The curves are then additionally smoothed out.

The circuit schematically shown in Fig. 4a is implemented to perform Hanbury–Brown–Twiss measurements^26^ using linear detectors^12^,^13^,^14^,^25^,^27^,^28^. We accumulate traces each consisting of a train of 40 pulses with a period of *T*=160 ns. The emitted photons are then transmitted through an isolator to a 90° hybrid coupler operating as a microwave beam splitter. The idle input port terminated by 50 Ω is a source of vacuum noise. The two signals coming from the splitter output ports of the hybrid coupler are amplified by amplifiers at 4.2 K and at room temperature. We assume that the noise added by the amplifiers are uncorrelated in each channel. Next, the signals, down-converted to close to zero-frequency with mixers, are passed through 30 MHz bandwidth low-pass filters and voltage amplitudes *V*_1_(*t*) and *V*_2_(*t*) are recorded by two digitisers. Finally, the traces are read out and treated by a PC to extract the two-point correlations functions.

First, we demonstrate the first-order correlation function 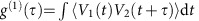 of the two signals. The normalized function with subtracted background 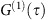 is exemplified in an inset of Fig. 4b. The central peak (*G*^(1)^(0)) corresponds to the total power emitted by the atom and the side peaks (*G*^(1)^(*nT*)) correspond to the coherent emission. The dynamics of the peaks as a function of the excitation pulse length is shown in Fig. 4b. The solid lines demonstrate simulations with the previously extracted device parameters.

Next, we calculate the second order correlated function of the emitted radiation defined as


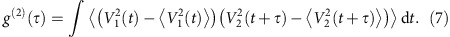


The result of 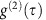 measurements after averaging of 1.5 × 10^10^ traces is shown in Fig. 4c, where the function 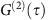 is obtained from 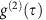 by subtracting the background and normalization. The traces are additionally smoothed to reduce fluctuations. The red curves show expectations calculated with the measured photon shapes. We observe a series of side peaks spaced by *T* with a suppressed peak at zero delay time 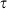
=0. The observed anti-bunching of the emission demonstrates the single-photon generation.

### Single-photon source efficiency at different frequencies

**Figure 5:**
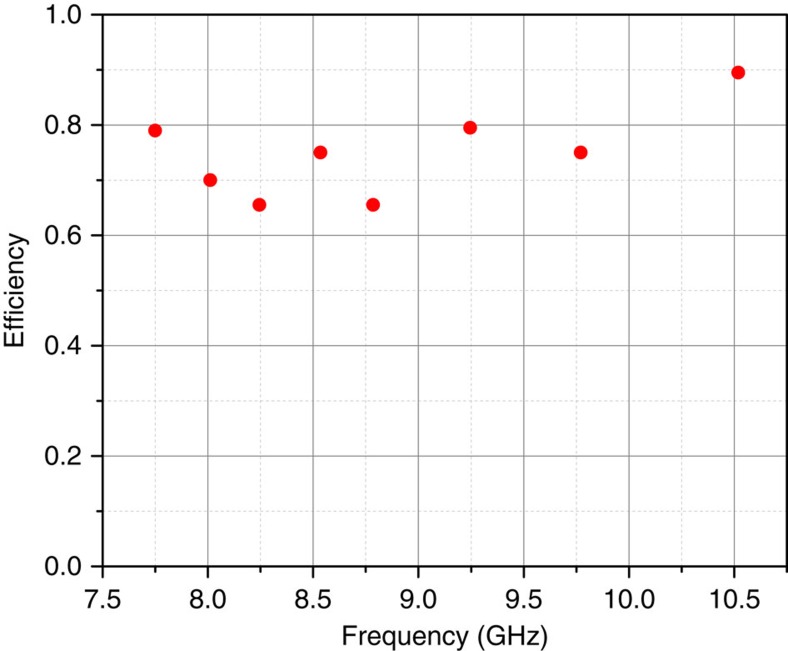
The single-photon source efficiency in a wide frequency range. The extracted efficiency of the photon source versus different transition frequencies of the two-level atom.

We evaluate the device-limited efficiency of our source over a wide frequency range by tuning the emission frequency *ω*_10_ controlled by *δ*Φ. First, we would like to point out that in our flux-qubit-based atom, pure dephasing 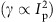 is expected to be strongly suppressed because of the low persistent current *I*_p_ being one order lower than in conventional designs^22^,^23^. Therefore, the pure dephasing should not affect the efficiency too much, even when we detune the energy from the minimal ones. We characterize the coupling strength using equation (5) by measuring the circle radius *r*_e_ at different frequencies in the complex plane, similar to Fig. 2b. Figure 5 shows the derived device intrinsically-limited efficiency as a function of frequency. We obtained more than 65% efficiency almost everywhere over the range of frequencies from 7.75 to 10.5 GHz. The efficiency can be affected by non-negligible pure dephasing *γ* and/or non-radiative relaxation 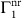.

We would like to point out that the efficiency of the excited state preparation in our experiments can be estimated as 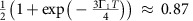 (ref. 19) reducing the total efficiency at the degeneracy point down to 0.79 × 0.87=0.69. This value is consistent with the one obtained after calibration of our setup, which is found to be 0.67.

## Discussion

In conclusion, we demonstrated an on-chip tuneable on-demand single-microwave-photon source operating with high efficiency over a wide range. The source is expected to be useful for applications including quantum communication, quantum information processing, and sensing.

### Data availability

The data that support the findings of this study are available from the corresponding author on request.

## Additional information

**How to cite this article:** Peng, Z. H. *et al*. Tuneable on-demand single-photon source in the microwave range. *Nat. Commun.* 7:12588 doi: 10.1038/ncomms12588 (2016).
